# Swedish matched cohort on the association between maternal polyendocrine metabolic ovarian syndrome and next-generation cancer risk

**DOI:** 10.1093/jncics/pkag104

**Published:** 2026-10-05

**Authors:** Romina Fornes, Unnur Gudnadottir, Sheila Orwa, Michael Saerens, Elisabet Stener-Victorin, Nele Brusselaers

**Affiliations:** Centre for Translational Microbiome Research (CTMR), Department of Microbiology, Tumor and Cell Biology, Karolinska Institutet, Stockholm, Sweden; Centre for Translational Microbiome Research (CTMR), Department of Microbiology, Tumor and Cell Biology, Karolinska Institutet, Stockholm, Sweden; Department of Women’s and Children’s Health, Karolinska Institutet, Stockholm, Sweden; Global Health Institute, Department of Family Medicine and Population Health, University of Antwerp, Antwerp, Belgium; Department of Medical Oncology, Ghent University Hospital, Ghent, Belgium; Cancer Research Institute Ghent, Ghent University, Ghent, Belgium; Department of Physiology and Pharmacology, Karolinska Institutet, Stockholm, Sweden; Centre for Translational Microbiome Research (CTMR), Department of Microbiology, Tumor and Cell Biology, Karolinska Institutet, Stockholm, Sweden; Department of Women’s and Children’s Health, Karolinska Institutet, Stockholm, Sweden

## Abstract

**Background:**

Polyendocrine metabolic ovarian syndrome (PMOS) is an endocrine and metabolic disorder associated with adverse perinatal and long-term health outcomes, yet its impact on cancer risk in children remains unclear.

**Methods:**

We conducted a nationwide, register-based matched cohort study in Sweden, including children of primiparous women with (*N* = 22 955) and without PMOS (*N* = 103 613) born between 1973 and 2018. Children were followed from birth until cancer diagnosis, death, or December 2018. Conditional Cox proportional hazards models estimated the adjusted hazard ratio (aHR) with 95% CI, incorporating time-varying effects when the proportional hazards assumption was violated.

**Results:**

During follow-up, 666 children were diagnosed with cancer. Compared with children born to women without PMOS, overall cancer risk among children of women with PMOS differed by sex and over time. Among sons, higher hazards at 1 year were observed (aHR = 4.17; 95% CI = 2.51 to 6.93), decreasing by 10 years (aHR = 1.53; 95% CI = 1.08 to 2.18). In daughters, hazard ratios did not indicate a change in overall cancer risk compared to reference. When both sexes were combined, risk of lymphatic and hematopoietic malignancies was higher (aHR = 2.43; 95% CI = 1.36 to 4.33). Competing risks of death did not materially alter the associations.

**Conclusions:**

Overall cancer risk among children born to women with PMOS differed by sex and over time, with higher risks in sons during early life. These findings suggest that maternal PMOS may be associated with differential cancer susceptibility across the life course.

## Introduction

Recent studies have revealed the potential impact of maternal metabolic conditions such as overweight, obesity, or diabetes on the risk of childhood malignancies, including leukemia and Wilms tumor.^1-4^ Emerging evidence further suggests that other maternal endocrine and obstetric conditions, including gestational hypertension and thyroid disorders, may also be associated with an increased risk of childhood cancer, particularly hematological malignancies.^5^^,^^6^ Together, these findings highlight the critical need to determine whether the risk of malignant tumors is similarly elevated in association with other maternal endocrine or metabolic conditions, particularly polyendocrine metabolic ovarian syndrome (PMOS, previously called polycystic ovary syndrome),^7^ a prevalent disorder affecting 10%-13% of women of reproductive age worldwide, and strongly linked to overweight and obesity.^8^ Women with PMOS often exhibit hyperandrogenemia, hyperinsulinemia, insulin resistance, and obesity, and have an elevated risk of gestational diabetes.^9-11^ Epidemiological evidence suggests that women with PMOS themselves have a higher risk of cancers of the endometrium, ovary, endocrine gland, pancreas, skeletal, and hematopoietic system.^12^ Regarding their daughters and sons, there is increasing information about the morbidity rate of infants, adolescents, and young adults born to a mother with PMOS,^13-16^ but limited information regarding cancer risks. To address this gap, it is reasonable to investigate whether children born to mothers diagnosed with PMOS might also be predisposed to developing malignant neoplasms. The aim of this study was to evaluate whether maternal PMOS is associated with overall and site-specific cancer risk in children from birth into adulthood.

## Methods

### Study design and data source

This research is a Swedish nationwide registry-based matched cohort study focusing on the children of women with PMOS. All women aged 14 and older with at least 1 recorded diagnosis of PMOS since 1964 were identified from the National Patient Register (NPR).^17^ From this group, all women with at least 1 pregnancy recorded in the Swedish Medical Birth Register (MBR)^18^ since 1973 were selected, and matched to 5 women who did not have the PMOS diagnosis, based on maternal age at first delivery, year of first delivery, and number of deliveries. Using the unique personal identification number assigned to all residents in Sweden, all the personal medical information recorded in the NPR, MBR, cancer register (CR), and cause of death register (DR) was linked. Finally, all biologically assigned female and male children born to primiparous mothers diagnosed with or without PMOS were included in this cohort and followed from birth until the first record of cancer diagnosis in the NPR or the CR, death or the end of the study (December 31, 2018), whichever occurred first. Pregnancies with multiple births, missing information about pregnancy length, no available identification number for newborns, no information about stillbirth, or matching sets without either case or control were excluded from the dataset (Figure S1). As the data were extracted by the Swedish national repository, no individual informed consent was required. The study was performed in accordance with the principles expressed in the Declaration of Helsinki, whereas the respective protocol was approved by the Swedish Ethical Review Authority (Dnr: 2019-00299, https://etikprovningsmyndigheten.se).^19^ Consequently, in accordance with national regulations, the data were accessed through the Swedish National Board of Health and Welfare (Socialstyrelsen; https://www.socialstyrelsen.se), who performed the data linkage and provided us with the linked and pseudonomised data as approved by the ethics committee.

### Exposure

Maternal PMOS was identified using the international classification of diseases (ICD) codes in the MBR of the Swedish versions ICD-10 (form 1997 onwards; E28.2), ICD-9 (1987-1997; 256E), and ICD-8 (1968-1986; 256,9) corresponding to the formerly known Stein-Leventhal syndrome. As PMOS is considered a lifelong condition, any record of the diagnosis in the registers at any time was included, regardless of its timing in relation to pregnancies.

### Outcomes

The risk of cancer was examined using 2 analytical approaches: an overall assessment of cancer risk and a site-specific analysis based on tumor anatomical locations, categorized according to the Swedish adaptations of ICD-8, ICD-9, and ICD-10 and the Coding of the Cancer Register, 2020^20^ (Table S1). Only the first malignant event ever recorded in either the CR or in the NPR (inpatient and outpatient) was included. Types of cancer were identified using the Swedish versions of ICD-10 (C00-C96) or ICD-7/8/9 (140-209). The specific ICD versions used to classify malignancies by anatomical group varied by registry: ICD-7 and ICD-10 codes were used in the CR; ICD-8, ICD-9, and ICD-10 were used in the NPR inpatient register (available since 1973), while only ICD-10 was used in the NPR outpatient register (available since 2001) (Table S1). All the codes used were confirmed in the working document for staff at regional cancer centers.^20^ An additional evaluation of the cancer classification was performed when the earlier diagnosis was identified in the NPR but was discordant with a later diagnose (within the following 30 days) in the CR. In those cases, the diagnose recorded in the CR was conserved (14 cases). Further, if the earlier record was identified in the NPR, but the register showed 2 diagnoses with 2 annotations of cancer in the same visit, but corresponded to different anatomical locations, then the main diagnosis was considered (5 cases).

### Covariates

The covariates included in the main analysis were selected from univariable analysis for overall cancer (*P* ≤ .1) and those that are considered clinically relevant and consistently available throughout the entire study period (1973-2018). These variables were categorized as follow: sex of the child (male/female), preterm birth before 37 weeks (yes/no), mode of delivery (cesarean section: yes/no), and maternal place of birth (Nordic/non-Nordic countries). Assisted reproduction was described as a baseline characteristic and evaluated in univariable analyses; however, it was not included in the main multivariable models due to incomplete availability before the mid-1990s, which could introduce differential misclassification across birth cohorts. Maternal body mass index (BMI) at enrollment (included in the model as <25, 25 to <30, ≥30 kg/m^2^) and any diabetes during pregnancy, both variables strongly related to PMOS, were excluded from the models in the main analysis due to incomplete availability across the study period. However, these were included in the models in the sub-analysis restricted to matching sets with the first delivery since 1992.

### Statistical analysis

In the present work, the study size was determined by the total number of eligible individuals identified in the nationwide Swedish registers. For all analyses, follow-up time was calculated from the birth until cancer diagnosis, death, or end of follow-up (December 31, 2018), whichever occurred first, except for acute lymphoblastic leukemia (ALL) (ICD-10: C910), where follow-up ended at age 10, death, or end of study, whichever occurred first. Although the original dataset contains all births from women with PMOS and matched controls, the main analysis in this study focused on first births, as the matching procedure was specifically implemented at first birth based on maternal age and year of delivery.

Only the first cancer diagnosis was included in the assessment of overall cancer risk and subsequently included in a separate analysis according to specific anatomical cancer location, restricted to cancer categories with more than 25 events. Incidence density rate (per 100 000 person-years) was calculated for overall and site-specific cancers. The association between maternal PMOS and cancer risk from birth into adulthood was assessed using multivariable conditional Cox proportional hazards models, stratified by matching set to account for the matched design. The covariates included child sex, cesarean section, preterm birth, and maternal place of birth.

For site-specific cancer analyses, the covariate adjustment strategy varied according to event frequency: for less frequent cancer types, models included child sex, cesarean section, and preterm birth; whereas for more frequent cancers maternal place of birth was additionally included. For female-specific cancers (C51-C58), adjustment for sex was omitted.

In these main analyses, the proportional hazards assumption was evaluated using the Schoenfeld residuals and visually by log-log Kaplan-Meier survival plots stratified by maternal PMOS status and offspring sex. When violated, time-dependent Cox regression models and interactions terms (if appropriate) were applied.

Subsequently, a sub-analysis restricted to matching sets with first delivery from 1992 onwards included additional adjustment for maternal BMI at enrollment and diabetes during pregnancy. Missing values for maternal BMI (<10%) were treated as a separate category. Time-dependent Cox regression models were also used in the sub-analysis when they had been applied in the main analysis, either for overall or site-specific cancer risk. Additional analyses using Fine-Gray subdistribution hazard models for overall cancer were performed to assess the potential impact of competing risks. In these analyses, cancer was treated as the event of interest and death from other causes as a competing event, while the same covariate set as in the main Cox models was included.

Results are reported as hazard ratios (HRs) and 95% CIs in both unadjusted (crude HR) and an adjusted model (adjusted HR, aHR). For Fine-Gray models, the results were reported as subdistribution HRs (sHRs) with 95% CIs for sons and daughters separately. Multicollinearity among covariates was assessed using variance inflation factors, and interactions between PMOS and covariates were evaluated using Wald and likelihood ratio tests; significant interactions (*P* < .05) were incorporated into the final models. All analyses were performed using R version 4.3.1 (survival package).^21^^,^^22^ This study is reported in accordance with the STROBE guidelines.

## Results

### Main analysis

A total of 22 955 (18%) children born to mothers with PMOS and 103 613 (82%) children born to mothers without PMOS were included (Figure S1). Maternal and pregnancy characteristics differed between the groups (Table 1). The PMOS group had a higher prevalence of maternal overweight (25.0% vs 19.9%) and obesity (19.7% vs 8.5%), a higher prevalence of preterm birth (8.4% vs 5.6%), cesarean sections (20.9% vs 17.1%), and maternal diabetes during pregnancy (4.1% vs 1.7%) compared to the non-PMOS group. The distribution of maternal age was similar between the groups (due to matching), with most women being between 25 and 30 years old at first delivery.

**Table 1.** Maternal and pregnancy characteristics of offspring born to primiparous mothers since 1973 and followed up to 2018, according to maternal PMOS diagnosis.

Variable	No-PMOS *n* (**%**)	PMOS *n* (%)
**Total**	103 613 (82)	22 955 (18)
**Maternal age, in years**		
**<25**	28 748 (27.7)	6055 (26.4)
**25 to <30**	38 767 (37.4)	8414 (36.7)
**30 to <35**	27 459 (26.5)	6302 (27.5)
**>35**	8639 (8.3)	2184 (9.5)
**Maternal BMI**		
**Underweight (<18.5 kg/m^2^)**	2913 (2.8)	320 (1.4)
**Normal (18.5-24.9 kg/m^2^)**	58 905 (56.9)	9685 (42.2)
**Overweight (25.0-29.9 kg/m^2^)**	20 568 (19.9)	5750 (25.0)
**Obese (≥30 kg/m^2^)**	8810 (8.5)	4529 (19.7)
**Missing**	12 417 (12.0)	2671 (11.6)
**Offspring sex**		
**Sons**	53 396 (51.5)	11 960 (52.1)
**Daughters**	50 217 (48.5)	10 995 (47.9)
**Preterm birth**		
**No**	97 827 (94.4)	21 023 (91.6)
**Yes**	5786 (5.6)	1932 (8.4)
**Cesarean section**		
**No**	85 874 (82.9)	18 165 (79.1)
**Yes**	17 739 (17.1)	4790 (20.9)
**Maternal place of birth**		
**Nordic countries**	83 350 (80.4)	17 755 (77.3)
**Other countries**	20 263 (19.6)	5200 (22.7)
**Any diabetes**		
**No**	101 851 (98.3)	22 007 (95.9)
**Yes**	1762 (1.7)	948 (4.1)
**Assisted reproduction**		
**No**	98 008 (94.6)	16 717 (72.8)
**Yes**	5605 (5.4)	6 238 (27.2)

Data shown as frequencies (*n*) and percentages (%).

Abbreviation: PMOS = polyendocrine metabolic ovarian syndrome.

### Cancer diagnosis

A total of 666 children were diagnosed with malignant cancer. Of these, 125 cases were among children born to mothers with PMOS, while 541 cases were among children born to mothers without PMOS. The incidence density rate was 53.5 and 50.6 per 100 000 person-years, respectively (Table 2). When analyzed separately by sex, the overall cancer incidence density rate was higher among daughters (PMOS: 64.3 vs No-PMOS: 74.8; per 100 000 person-years) than among sons (PMOS: 43.6 vs No-PMOS: 27.6; per 100 000 person-years) (Table 2, Figure S2). Crude incidence curves by sex for the main cohort are shown in Figure S3.

**Table 2.** Cancer incidence and person-time rates by anatomical site in primiparous offspring born between 1973 and followed up to 2018, according to presence or absence of maternal PMOS.

Cancer type	Group	Total (*n*)	Events (*n*)	**Incidence density rate (per 100** **000 person-years)**
**Overall cancer (both sex)**				
	No-PMOS	103 613	541	50.6
	PMOS	22 955	125	53.5
**Overall cancer female**				
	No-PMOS	50 217	389	74.8
	PMOS	10 995	72	64.3
**Overall cancer male**				
	No-PMOS	53 396	152	27.6
	PMOS	11 960	53	43.6

Abbreviations: PMOS = polyendocrine metabolic ovarian syndrome; *n* = frequency.

When considering both sexes combined, among cancer types with the highest cumulative incidences, the most frequent cancers in the PMOS group included lymphohematopoietic neoplasms (C81-C96, excluding ALL), which accounted for 14.4%, ALL (C910) (11.2%), and cancers of the central nervous system (C69-C72) (14.4%) compared with 6.5%, 10.7%, and 11.3%, respectively, in the No-PMOS group. In contrast, melanomas (C43-C44) and female genital organ neoplasms (C51-C58) were more common in the No-PMOS group, representing 8.5% and 43.1% of cases, compared with 4.8% and 31.2% in the PMOS group, respectively (Table S2).

Overall, 542 offspring died during the entire follow-up, including 143 of 22 955 (0.62%) individuals born to mothers with PMOS and 399 of 103 613 (0.39%) born to mothers without PMOS. Of these deaths, 39 occurred after a cancer diagnosis, whereas 503 occurred in individuals without prior recorded cancer. These low mortality proportions suggest that overall mortality is unlikely to have a major impact on the estimated cancer incidence.

### Prenatal exposure to maternal PMOS and cancer risk

In the main analysis, which included all first deliveries since 1973, both the crude and the adjusted models assessing the association between maternal PMOS and overall cancer risk in children showed a violation of the proportional hazards assumption in the global Schoenfeld test (*P* < .001), mainly driven by the variables offspring sex (*P* < .001) and main exposure (PMOS) (*P* < .05). Subsequently, interaction terms between PMOS and the covariates were tested. A significant interaction was found with offspring sex (*P* = .006), while other interactions were not statistically significant. Therefore, the final model was fitted using a time-varying Cox regression that included both the PMOS × offspring sex interaction and a sex-specific time-dependent PMOS effect. Under this model, sons of mothers with PMOS compared to those without PMOS showed an increased risk for overall cancer in the first year of follow-up [aHR = 4.17 (95% CI = 2.51 to 6.93)] decreasing to [aHR = 1.53 (95% CI = 1.08 to 2.18)] at 10 years. Among daughters, lower hazards were observed in those born to mothers with PMOS compared to those without PMOS, but remained non-significant [aHR = 0.88 (95% CI = 0.52 to 1.50), at 1 year] and [aHR = 0.83 (95% CI = 0.55 to 1.25)] at year 45 (Figure 1 and Table S3). The unadjusted cumulative incidence curve is shown in Figure S4, and univariable HRs in Table S4.

**Figure 1.**
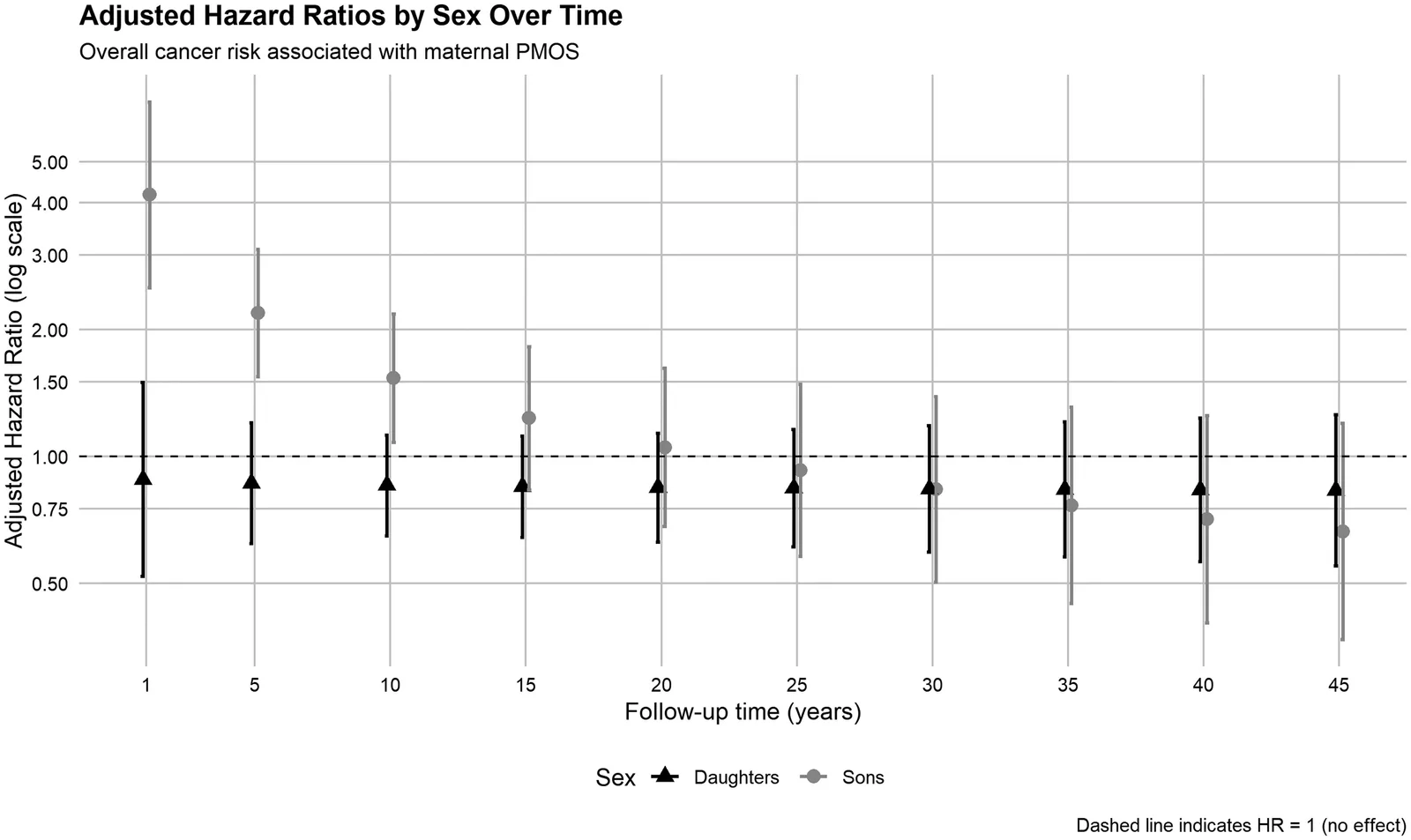
Time-varying adjusted hazard ratios for overall cancer in offspring of mothers with PMOS, by offspring sex. Hazard ratios were estimated from conditional Cox regression models including a time-dependent effect for PMOS and an interaction between PMOS and offspring sex, adjusted for cesarean section, preterm birth (<37 weeks), and maternal country of birth (Nordic vs non-Nordic), and stratified by matching set. Abbreviations: HR = hazard ratio; PMOS = polyendocrine metabolic ovarian syndrome.

In supplementary competing-risks analyses, maternal PMOS remained associated with an increased sHR of overall cancer among sons [sHR = 1.53 (95% CI = 1.11 to 2.09)], while the corresponding estimate for daughters the results were not statistically significant [sHR = 0.83 (95% CI = 0.64 to 1.07)]. These results were similar to those observed in the cause-specific Cox models suggesting that competing risks of death do not alter the sex-specific associations between maternal PMOS and overall cancer in offspring.

For site-specific cancer analyses, conventional conditional Cox models were used, with offspring sex included as an adjustment variable rather than as a stratification factor, as no evidence of non-proportional hazards or interaction with sex was observed for these outcomes and the limited number of cases did not support further sex-stratified modeling. Accordingly, maternal PMOS was associated with an increased risk of lymphatic and hematopoietic malignancies (excluding ALL) in children, compared to those born to mothers without PMOS [aHR = 2.43 (95% CI = 1.36-4.33)], and an incidence rate of 7.7 vs 3.3 per 100 000 person-years in exposed and unexposed children, respectively (Table 3). The analysis of the value of this association showed an *E*-value of 4.29 for the point estimate, and the *E*-value for the lower bound of the 95% CI is 2.06. Cumulative incidence curves (unadjusted) are shown in Figure S4 and the frequency of the subcategories of lymphatic and hematopoietic cancers (excluding ALL) is presented in Table S5.

**Table 3.** Association between maternal PMOS and site-specific cancer risk in offspring born since 1973 and followed up to diagnosis, death, or up to December 2018.

Cancer type	No-PMOS/PMOS	Total	**Incidence density rate (per 100** **000 person-years)**	Events	HR crude	aHR (95% CI)^a^
**Melanoma and other skin**						
	No-PMOS	103 613	4.3	46	Ref	Ref
	PMOS	22 955	2.6	6	0.58 (0.25-1.36)	0.51 (0.24-1.40)
**Female genital organs^b^**						
	No-PMOS	24 190	48.0	120	Ref	Ref
	PMOS	10 372	36.8	39	0.76 (0.53-1.11)	0.77 (0.53-1.12)
**Eye, brain and other parts of CNS**						
	No-PMOS	103 613	5.7	61	Ref	Ref
	PMOS	22 955	7.7	18	1.32 (0.78-2.24)	1.29 (0.75-2.20)
**Lymphatic, hematopoietic (excluding ALL)**						
	No-PMOS	103 613	3.3	35	Ref	Ref
	PMOS	22 955	7.7	18	**2.42 (1.36-4.29)**	**2.43 (1.36-4.33)**
**ALL^c^**						
	No-PMOS	103 613	7.5	53	Ref	Ref
	PMOS	22 955	7.0	11	0.95 (0.49-1.82)	1.00 (0.51-1.96)

Abbreviations: aHR = adjusted hazard ratio; ALL = acute lymphoblastic leukemia; CNS = central nervous system; HR = hazard ratio; PMOS = polyendocrine metabolic ovarian syndrome; Ref = reference.

a Adjusted by sex of child, cesarean section, preterm birth (<37 weeks), and maternal country of birth. All models were stratified by matching set and estimated using conventional conditional Cox proportional hazards models.

b Only matching sets that had at least 1 female offspring in mothers without PMOS (male offspring were removed from the analysis).

c Follow-up to 10 years of age.

Values in bold indicate statistical significance.

### Sub-analysis

To account for the availability of the additional metabolic covariates, the sub-analysis included children born to primiparous mothers in or after 1992 (*n* = 118 793). Consistent with the main analysis, the assessment of overall cancer showed that only sons in the PMOS group had increased hazards compared with sons in the non-PMOS group. In Model 1, which retained the time-dependent specification and interaction structure used in the main cause-specific analysis (including the PMOS × sex interaction and the time-varying PMOS effect), the aHR was 2.42 (95% CI = 1.41 to 4.15) at 1 year and 1.65 (95% CI = 1.11 to 2.44) at 5 years. Additional adjustment for maternal BMI (Model 2) showed an increased risk only among sons at 1 year [aHR = 2.38 (95% CI = 1.38 to 4.11)] and [aHR = 1.63 (95% CI = 1.09 to 2.43)] at 5 years. Further adjustment by any diabetes during pregnancy (Model 3) showed similar results [aHR = 2.38 (95% CI = 1.38 to 4.11)] at 1 year and [aHR = 1.63 (95% CI = 1.09 to 2.43)] at 5 years (Table S6).

For the assessment of malignant cancer by specific anatomical locations, analyses were conducted with sons and daughters combined. This approach was applied because no evidence of sex interaction or non-proportional hazards was observed for these outcomes, and the number of events did not support sex-stratified analyses. Accordingly, an elevated risk of lymphatic and hematopoietic malignancies (excluding ALL) was observed [aHR = 3.25 (95% CI = 1.65 to 6.43)]. Similar results were observed when maternal BMI at the beginning of pregnancy was included as a covariate (Model 2) and when both maternal BMI and maternal diabetes were included (Model 3) [aHR = 3.25 (95% CI = 1.65 to 6.43)] (Table S7).

## Discussion

In this large nationwide register-based matched cohort study including over 126 568 individuals born to primiparous mothers followed for up to 1 302 797 person-years, we found that maternal PMOS was associated with sex- and time-dependent differences in overall cancer risk in offspring. Specifically, higher hazards were observed in sons during early life, and this excess risk attenuated as they aged, whereas findings in daughters born to mothers with PMOS did not indicate a clear change in overall cancer risk compared with the reference group. In addition, an increased risk of lymphatic and hematopoietic malignancies was identified when both sexes were analyzed together.

One of the main strengths of this study is its large sample size and the use of high-quality nationwide register data, which enhance the validity of the findings, particularly for rare outcomes. This matched cohort included 126 568 children born to primiparous mothers, with follow-up of up to 45 years. The use of relevant clinical variables reduced the risk of selection bias. In a sub-analysis restricted to births from 1992 onwards, additional adjustment for maternal BMI and diabetes during pregnancy was possible, further strengthening the observed associations.

Several limitations merit consideration. Incomplete data for key covariates, particularly maternal BMI and diabetes status, may limit the generalizability of findings. Residual confounding by maternal metabolic factors cannot be excluded due to incomplete data availability, particularly for earlier birth cohorts. Despite the large sample size, the number of events for specific cancer subtypes was limited, reducing statistical power for some analyses. Consequently, our site-specific analyses, particularly those based on very few events, should be regarded as exploratory and hypothesis-generating, and the possibility of chance findings, including type I error due to multiple comparisons, cannot be excluded. As follow-up extends into later adulthood, survivor and other selection mechanisms may still contribute to the attenuation of HRs and should be considered when interpreting the long-term patterns, particularly in daughters. Furthermore, reliance on registry-based diagnoses may have led to misclassification of PMOS exposure due to changes in ICD coding of PMOS and diagnostic awareness over time, as well as preferential inclusion of either milder or more severe PMOS cases, potentially affecting generalizability. Because diagnostic awareness and coding of PMOS were more limited in earlier calendar periods, under-reporting and exposure misclassification are likely to have been more pronounced among women diagnosed in the oldest eras, which may have diluted some associations and contributed to the attenuation of long-term effects observed in our time-varying models. Lastly, surveillance bias due to differential healthcare contact may also have influenced the observed associations.

To address the possibility that differential mortality might distort the observed associations, we additionally explored the potential role of competing risks by performing Fine-Gray subdistribution hazard analyses for overall cancer. Similarly to cause-specific Cox estimates, the Fine-Gray results showed an excess risk in sons and no statistically significant difference in daughters compared with the non-exposed group. Taken together, these findings suggest that competing risks of death are unlikely to be the primary driver of the observed sex differences in cancer risk.

As previously mentioned, studies have reported that children born to mothers with PMOS have increased hospitalization rate for metabolic disorders, neurological diseases, and asthma, highlighting the possible adverse impact of maternal metabolic conditions on childhood health.^16^ However, evidence on the risk of cancer in the offspring of women with PMOS remains very limited. Only one large population-based study in Canada of 1 038 375 children (7160 born to mothers with PMOS) followed for up to 13 years directly assessed the association between PMOS and cancer in offspring and found no significant increase in cancer risk before age 13. However, this study analyzed only severe childhood morbidity requiring hospitalization. Therefore, those results could have been affected by the characteristics of the data and the small number of children born to mothers with PMOS who developed cancer (only 12 cases).^23^

In the absence of prior literature specifically addressing cancer risk in children of mothers with PMOS, it is important to evaluate studies of other maternal conditions with metabolic alterations similar to PMOS. Maternal adverse conditions, such as pregestational or gestational diabetes and maternal obesity, have been linked to increased risk of cancer in offspring, especially childhood leukemia and other hematological malignancies.^24-27^ A recent publication studying all the pregnancies in Sweden since 1983, found that the maternal BMI ≥25 kg/m^2^ was associated with an increased risk of leukemia in daughters, but not in sons.^27^ Thus, our findings of a higher risk of cancer—particularly lymphoid and hematological neoplasms, in children of women with PMOS are consistent with patterns reported for other maternal metabolic disorders, supporting the hypothesis that endocrine or metabolic disturbances during pregnancy may have long-term oncologic consequences for children. In the sub-analysis restricted to births from 1992 onwards, additional adjustment for maternal BMI at the first antenatal visit and diabetes during pregnancy did not abolish the excess early-life risk in sons. Because BMI measured around gestational week 10 is a crude proxy of the relevant metabolic and inflammatory milieu, we regard these BMI-adjusted models as sensitivity analyses rather than definitive evidence against a metabolic contribution to the observed associations.

Beyond maternal metabolic factors, reproductive conditions also warrant consideration as potential confounders, particularly the use of assisted reproductive technologies in women with PMOS. According to previous reports, PMOS accounts for approximately 80% of women with anovulatory infertility.^28^ In a large community-based cohort, infertility was reported in 72% of women with PMOS compared with 16% of women without PMOS, substantially greater use of fertility hormone treatments among women with PMOS.^29^ These patterns are consistent with the higher proportion of ART use observed among mothers with PMOS in our cohort (27.2% vs 5.4% in the non-PMOS group). However, ART was not registered before 1994. After that period, only 28 sons or daughters who developed cancer were conceived with ART. Because of the few events, ART was excluded from the main multivariable model. We therefore analyzed the variable restricted to births after 1992 (although no ART records were available between 1992 and 1994). In a secondary sensitivity analysis, of overall cancer risk including maternal BMI, maternal diabetes, an increased risk was observed only among sons at 1 year of follow-up [aHR = 2.10 (95% CI = 1.18 to 3.71)] (results not shown), whereas the association was not statistically from the reference thereafter. Of note, infertility treatments were grouped all of them into a single variable considering IVF/ICSI, together with non-ART interventions (ovulation stimulation, surgery, and other procedures) which limits our ability to isolate the specific effect of ART on offspring cancer risk.

In this study, we found an increased risk of hematopoietic and lymphatic malignancies (excluding ALL) in offspring of mothers with PMOS. The robustness of this association was supported by the *E*-value analysis, which indicated that only moderately strong to strong unmeasured confounders would be capable of substantially attenuating the association. Evidence on the role of maternal androgens in carcinogenesis remains limited. Importantly, more recent studies suggest that androgens may influence immune regulation rather than acting directly on malignant transformation. Experimental models of PMOS have demonstrated that androgen excess alters immune cell composition and activation, including modulation of B-cell–mediated immune responses.^30^^,^^31^ These alterations appear to be mediated through androgen receptor-dependent mechanisms rather than a single immune pathway. Given the central role of immune regulation in hematological malignancies, these findings raise the possibility that prenatal or early-life exposure to altered androgenic and immunometabolic environments could indirectly influence cancer susceptibility through immune-mediated mechanisms. Further mechanistic and translational studies are needed to clarify whether such immune alterations contribute to cancer risk later in life.

Another plausible biological mechanism linking maternal PMOS to cancer in offspring involves alterations in the maternal microbiota. Population-based studies have shown that offspring of mothers with PMOS have an increased risk of infectious diseases during childhood, suggesting altered immune function early in life.^23^ In parallel, women with PMOS exhibit increased susceptibility to infections and are more likely to use antibiotics compared with those without PMOS.^32^ These findings support the presence of broader immune dysregulation in PMOS that may affect offspring. In this context, the maternal gut microbiota—known to be altered in PMOS^33^—and microbial signaling pathways, which can influence early-life immune development,^34^ may represent additional mechanisms linking maternal immunometabolic disturbances with fetal programming. Although direct evidence linking these mechanisms to cancer risk is lacking, such early immune perturbations may contribute to long-term disease susceptibility.

Women with PMOS are already recognized as a high-risk group for obstetric and neonatal complications. Our findings extend this perspective by suggesting that maternal PMOS may also have long-term implications for offspring health, including differences in cancer risk across the life course. These associations appear to vary by sex and age, highlighting the importance of developmental timing and sex-specific mechanisms. While these findings reflect population-level associations rather than individual risk, they underscore the need to better characterize PMOS phenotypes and to determine whether improved metabolic and endocrine management before or during pregnancy could modify these long-term outcomes.

## Conclusion

In this nationwide cohort study, maternal PMOS was associated with time- and sex-dependent differences in overall cancer risk, with higher hazards in sons at early ages. In addition, an increased risk of lymphatic and hematopoietic malignancies was observed when both sexes were analyzed together. Although these findings should be interpreted as associations rather than causal effects, they highlight the potential long-term impact of maternal PMOS on offspring health. Further studies integrating metabolic, endocrine, and immune pathways are needed to clarify underlying mechanisms and inform potential preventive strategies.

## Supplementary Material

pkag104_Supplementary_Data

## Data Availability

Data are not openly available due to the sensitive personal nature of it and Swedish data regulations. Researchers may apply for data access through the appropriate Swedish authorities. Code used for data analysis is available on a reasonable request.
