# Supplementary material for: Swedish matched cohort on the association between maternal polyendocrine metabolic ovarian syndrome and next-generation cancer risk
Source: JNCI Cancer Spectr. 2026 Oct 5;10(5):pkag104. doi: 10.1093/jncics/pkag104 (PMC13718594; doi:10.1093/jncics/pkag104)
Supplement: Supplementary file 1 [file pkag104_supplementary_data.docx]

**Supplementary material**

Figures

1. Figure S1. Inclusion and exclusion criteria of study matched cohort.
2. Figure S2. Incidence rates of overall cancer among boys (left) and girls (right) born to mothers with and without Polyendocrine Metabolic Ovarian Syndrome (PMOS).
3. Figure S3. Log–log Kaplan–Meier survival curves for overall cancer by maternal PMOS status and offspring sex
4. Figure S4. Cumulative incidence for overall cancer (left) and Lymphatic, hematopoietic

and related tissue excluding acute lymphoblastic leukemia (right).

Tables

1. Table S1. International Classification of Disease (ICD) codes used to classify outcomes.
2. Table S2. Frequencies of cancer according to anatomical location in main analysis.
3. Table S3. Univariable hazard ratios and 95% Confidence Interval (CI) for maternal and perinatal factors associated with overall cancer risk in offspring.
4. Table S4. Time-varying hazard ratios for overall cancer in offspring of mothers with Polyendocrine Metabolic Ovarian Syndrome (PMOS), by offspring sex.
5. Table S5. Frequency of Cancer Category C81-C96 (excluding C910, Acute lymphoblastic leukemia)
6. Table S6. Time-dependent sub-analysis of the association between maternal polyendocrine Metabolic Ovarian Syndrome (PMOS) and overall cancer risk among sons born since 1992, with follow-up until diagnosis, death, or December 2018.
7. Table S7. Sub-analysis of the association between maternal Polyendocrine Metabolic Ovarian Syndrome (PMOS) and site-specific cancer risk in offspring born since 1992, followed up until diagnosis, death, or December 2018.

**Figure S1. Inclusion and exclusion criteria of study matched cohort.**

**Figure S2. Incidence rates of overall cancer among boys (left) and girls (right) born to mothers with and without Polyendocrine Metabolic Ovarian Syndrome (PMOS).**

Abbreviations: PMOS: Polyendocrine Metabolic Ovarian Syndrome

**Figure S3. Log–log Kaplan–Meier survival curves for overall cancer by maternal Polyendocrine Metabolic Ovarian Syndrome (PMOS) status and offspring sex.**

**Figure S4. Cumulative incidence for overall cancer (left) and Lymphatic, hematopoietic and related tissue excluding acute lymphoblastic leukemia (right) for children born to women with or without Polyendocrine Metabolic Ovarian Syndrome (PMOS).**

**Table S1. International Classification of Disease (ICD) codes used to classify outcomes.**

| Type of malignant neoplasms | ICD10 | ICD9 | ICD8 | ICD7 |
| --- | --- | --- | --- | --- |
| Overall (first cancer, excluding cancers of female and male genital organs) | C00-C96 (excl.C51-63) | 140 to 208x (excl.179-187x) | 140 to 209 (excl.180 -187) | 140 to 209 (excl.171-179) |
| Lip, oral cavity and pharynx | C00-C14 | 140 to 149x | 140 to 149 | 140 to 148 |
| Digestive system | C15-C26 | 150 to 159x | 150 to 159 | 150 to 158 |
| Respiratory system and thoracic cavity | C30-C39 | 160 to 165x | 160 to 163 | 160-164 |
| Bone and articular cartilage | C40-C41 | 170 to 170x | 170 | 196 |
| Melanoma and other skin | C43-C44 | 172 to 173x | 172 to 1739 | 190 ; 191 |
| Mesothelial (body cavity-lining) tissue and soft tissue | C45-C49 | 171 to 171x | 171 to 1719 | 1970 to 1979; 1933; 1939 |
| Malignant neoplasm of breast | C50-C50 | 174 to 175 | 174 | 170 |
| Female genital organs | C51-C58 | 179 to 184x | 180 to 184 | 171 to 176 |
| Male genital organs | C60-C63 | 185 to 187x | 185 to 187 | 177 to 179 |
| Urinary system | C64-C68 | 1880 to 189x | 188 to 189 | 180; 181 |
| Eye, brain and other parts of central nervous system | C69-C72 | 190 to 192x | 190 to 1929 | 192; 1930 |
| Thyroid and other endocrine glands | C73-C75 | 193 to 194x | 193 to 194 | 194 to 195 |
| Incompletely specified, secondary and unspecified sites | C76-C80 | 195 to 199 | 195 to 199 | 199 |
| Lymphatic, hematopoietic (excl. Lymphatic leukemia) | C81-C90 C911-C96 | 200 to 203;  204B to 208x | 200 to 203,99; 204,15 to 209,9 | 200 to 203; 2041;2049; 205 to 209 |
| Lymphatic leukemia | C910 | 204a | 204,00 | 204 |

Abbreviations: ICD: International Classification of Diseases.

**Table S2. Frequencies of cancer according to anatomical location in main analysis.**

Abbreviation= PMOS: Polyendocrine Metabolic Ovarian Syndrome ; ALL: Acute lymphoblastic leukemia (ALL).

| Type of malignant neoplasms | | No-PMOS  n (%) | PMOS  n (%) |
| --- | --- | --- | --- |
| Overall Cancer | First cancer identified | 541 (82) | 125 (18) |
| C00_C14 | Lip, oral cavity and pharynx | 8 (1.5) | 5 (4) |
| C15_C26 | Digestive system | 12 (2.2) | 5 (4) |
| C30_C39 | Respiratory system and thoracic cavity | 7 (1.3) | 0 (0) |
| C40_C41 | Bone and articular cartilage | 8 (1.5) | 2 (1.6) |
| C43_C44 | Melanoma and other skin | 46 (8.5) | 6 (4.8) |
| C45_C49 | Mesothelial (body cavity-lining) tissue and soft tissue | 23 (4.3) | 3 (2.4) |
| C50_C50 | Malignant neoplasm of breast | 7 (1.3) | 1 (0.8) |
| C51_C58 | Female genital organs | 233 (43.1) | 39 (31.2) |
| C60_C63 | Male genital organs | 10 (1.8) | 4 (3.2) |
| C64_C68 | Urinary system | 17 (3.1) | 4 (3.2) |
| C69_C72 | Eye, brain and other parts of central nervous system | 61 (11.3) | 18 (14.4) |
| C73_C75 | Thyroid and other endocrine glands | 13 (2.4) | 4 (3.2) |
| C76_C80 | Incompletely specified, secondary and unspecified sites | 3 (0.6) | 2 (1.6) |
| C81_ C96 (excl. ALL) | Lymphatic, hematopoietic and related tissue (excl. ALL) | 35 (6.5) | 18 (14.4) |
| C910 | Acute Lymphatic leukemia (ALL) | 58 (10.7) | 14 (11.2) |

**Table S3. Time-varying hazard ratios for overall cancer in offspring of mothers with Polyendocrine Metabolic Ovarian Syndrome (PMOS), by offspring sex**

| Years | Sons | | Daughters | |
| --- | --- | --- | --- | --- |
|  | **Crude HR (CI 95%)** | **aHR (CI 95%)** | **Crude HR (CI 95%)** | **aHR (CI 95%)** |
|  |  |  |  |  |
| 1 | **4.13 (2.49-6.86)** | **4.17 (2.51-6.93)** | 0.87 (0.51-1.48) | 0.88 (0.52-1.5) |
| 5 | **2.2 (1.55-3.11)** | **2.19 (1.55-3.1)** | 0.86 (0.62-1.19) | 0.86 (0.62-1.2) |
| 10 | **1.55 (1.09-2.2)** | **1.53 (1.08-2.18)** | 0.85 (0.65-1.12) | 0.85 (0.65-1.12) |
| 15 | 1.25 (0.85-1.84) | 1.23 (0.83-1.82) | 0.85 (0.64-1.12) | 0.85 (0.64-1.12) |
| 20 | 1.07 (0.69-1.65) | 1.05 (0.68-1.62) | 0.84 (0.63-1.13) | 0.84 (0.63-1.13) |
| 25 | 0.95 (0.59-1.51) | 0.93 (0.58-1.48) | 0.84 (0.61-1.16) | 0.84 (0.61-1.16) |
| 30 | 0.85 (0.52-1.42) | 0.84 (0.5-1.39) | 0.84 (0.59-1.18) | 0.84 (0.59-1.18) |
| 35 | 0.78 (0.46-1.34) | 0.77 (0.45-1.31) | 0.84 (0.58-1.21) | 0.83 (0.58-1.21) |
| 40 | 0.73 (0.41-1.28) | 0.71 (0.4-1.25) | 0.84 (0.56-1.23) | 0.83 (0.56-1.23) |
| 45 | 0.68 (0.38-1.23) | 0.66 (0.37-1.2) | 0.83 (0.55-1.26) | 0.83 (0.55-1.25) |

Abbreviations= PMOS, Polyendocrine Metabolic Ovarian Syndrome; HR, hazard ratio; aHR, adjusted hazard ratio; CI, confidence interval.
Hazard ratios were estimated from conditional Cox regression models including a time-dependent effect for PMOS and an interaction between PMOS and offspring sex, adjusted for cesarean section, preterm birth (<37 weeks), and maternal country of birth (Nordic vs. non-Nordic), and stratified by matching set.

**Table S4. Univariable hazard ratios and 95% Confidence Interval (CI) for maternal and perinatal factors associated with overall cancer risk in offspring.**

| Variable | HR_CI | P_value |
| --- | --- | --- |
| Polyendocrine metabolic ovarian syndrome | HR: 1.08 (95%CI: 0.89-1.31) | p = 0.453 |
| Underweight | HR: 1.17 (95%CI: 0.77-1.77) | p = 0.452 |
| Overweight | HR: 1.07 (95%CI: 0.84-1.34) | p = 0.594 |
| Obese | HR: 1.08 (95%CI: 0.77-1.51) | p = 0.650 |
| missing BMI | **HR: 0.73 (95%CI: 0.59-0.89)** | **p = 0.002** |
| Small for gestational age | HR: 0.99 (95%CI: 0.67-1.47) | p = 0.955 |
| Large for gestational age | HR: 1.38 (95%CI: 0.85-2.24) | p = 0.198 |
| Girl | **HR: 2.39 (95%CI: 2.03-2.82)** | **p < 0.001** |
| Preterm <37 weeks | HR: 0.87 (95%CI: 0.62-1.21) | p = 0.399 |
| C-Section | HR: 1.18 (95%CI: 0.96-1.45) | **p = 0.107** |
| maternal Nordic countries | HR: 0.89 (95%CI: 0.71-1.11) | p = 0.316 |
| Diabetes pregestational | HR: 0.77 (95%CI: 0.28-2.07) | p = 0.602 |
| Gestational diabetes | HR: 1.53 (95%CI: 0.76-3.08) | p = 0.235 |
| Any diabetes | HR: 1.14 (95%CI: 0.62-2.07) | p = 0.673 |
| Assisted reproduction | **HR: 1.61 (95%CI: 1.14-2.27)** | **p = 0.007** |
| Apgar <7 at 5 minutes | HR: 0.78 (95%CI: 0.37-1.64) | p = 0.515 |
| Tobacco at enrollment | HR: 1.12 (95%CI: 0.93-1.35) | p = 0.215 |

**Table S5. Frequency of Cancer Category C81-C96 (excluding C910, Acute lymphoblastic leukemia)**

| **Cancer Category** | **Total**  **Frequency** | **Frequency**  **No-PMOS** | **Frequency PMOS** |
| --- | --- | --- | --- |
|  | **53** | **35** | **18** |
| C81.0 Nodular lymphocyte-dominant Hodgkin lymphoma | 1 (1.9) | 1 (2.9) | 0 (0) |
| C81.1 Hodgkin lymphoma (classical) with nodular sclerosis | 8 (15.1) | **4 (11.4)** | **4 (22.2)** |
| C81.2 Hodgkin lymphoma (classical) with mixed cellularity | 3 (5.7) | 1 (2.9) | 2 (11.1) |
| C81.9 Hodgkin lymphoma, unspecified | 2 (3.8) | 1 (2.9) | 1 (5.6) |
| C82.9 Follicular lymphoma, unspecified | 1 (1.9) | 1 (2.9) | 0 (0) |
| C83.3 Diffuse large B-cell lymphoma | 1 (1.9) | 1 (2.9) | 0 (0) |
| C83.7 Burkitt's lymphoma | 3 (5.7) | 1 (2.9) | 2 (11.1) |
| C83.8 Other non-follicular lymphomas | 1 (1.9) | 1 (2.9) | 0 (0) |
| C84.5 Other mature T/NK cell lymphoma | 1 (1.9) | 1 (2.9) | 0 (0) |
| C85.9 Non-Hodgkin lymphoma, unspecified type | 1 (1.9) | 1 (2.9) | 0 (0) |
| C88.7 Other specified malignant immunoproliferative diseases | 1 (1.9) | 1 (2.9) | 0 (0) |
| C90.0 Multiple myeloma | 1 (1.9) | 1 (2.9) | 0 (0) |
| C90.2 Extramedullary plasmacytoma | 1 (1.9) | 1 (2.9) | 0 (0) |
| C92.0 Acute myeloid leukemia [AML] | 10 (18.9) | **7 (20)** | **3 (16.7)** |
| C92.1 Chronic myeloid leukemia [CML], BCR/ABL positive | 2 (3.8) | 2 (5.7) | 0 (0) |
| C92.4 Acute promyelocytic leukemia [PML] | 1 (1.9) | 1 (2.9) | 0 (0) |
| C92.5 Acute myelomonocytic leukemia | 1 (1.9) | 1 (2.9) | 0 (0) |
| C93.7 Other specified monocyte leukemia | 1 (1.9) | 0 (0) | 1 (5.6) |
| C94.0 Acute erythroid leukemia | 1 (1.9) | 1 (2.9) | 0 (0) |
| C94.2 Acute megakaryoblastic leukemia | 1 (1.9) | 1 (2.9) | 0 (0) |
| C94.6 Myelodysplastic and myeloproliferative disease, unclassified | 1 (1.9) | 1 (2.9) | 0 (0) |
| C95.0 Acute leukemia of unspecified cell type | 2 (3.8) | 1 (2.9) | 1 (5.6) |
| C95.9 Leukemia, unspecified | 2 (3.8) | 1 (2.9) | 1 (5.6) |
| C96.0 Multifocal and multisystemic (disseminated) Langerhans cell histiocytosis [Letterer-Siwes disease] | 1 (1.9) | 0 (0) | 1 (5.6) |
| C96.2 Malignant mast cell tumor | 1 (1.9) | 1 (2.9) | 0 (0) |
| C96.5 Multifocal and unisystemic Langerhans cell histiocytosis | 2 (3.8) | 2 (5.7) | 0 (0) |
| C96.6 Unifocal Langerhans cell histiocytosis | 2 (3.8) | 0 (0) | 2 (11.1) |

Abbreviation: ALL: Acute lymphoblastic leukemia (ALL)

**Table S6. Time-dependent sub-analysis of the association between maternal Polyendocrine Metabolic Ovarian Syndrome (PMOS) and overall cancer risk among sons born since 1992, with follow-up until diagnosis, death, or December 2018.**

| Follow-up time | Model 1 aHR (95% CI) | Model 2 aHR (95% CI) | Model 3 aHR (95% CI) |
| --- | --- | --- | --- |
| 1 year | 2.42 (1.41–4.15) | 2.38 (1.38–4.11) | 2.38 (1.38–4.11) |
| 5 years | 1.65 (1.11–2.44) | 1.63 (1.09–2.43)) | 1.63 (1.09–2.43) |

Abbreviations= PMOS: Polyendocrine Metabolic Ovarian Syndrome; HR: Hazard ratio; aHR: adjusted Hazard ratio; CI: confidence interval;
Model 1: Adjusted for offspring sex, cesarean section, preterm birth (<37 weeks), and maternal country of birth (Nordic vs. non-Nordic); included an interaction term between PMOS and offspring sex; stratified by matching set. Results are shown for male offspring only.
Model 2: Model 1 plus maternal BMI at enrollment.
Model 3: Model 2 plus any maternal diabetes during pregnancy.
Hazard ratios were estimated using time-dependent conditional Cox regression models.

**Table S7. Sub-analysis of the association between maternal Polyendocrine Metabolic Ovarian Syndrome (PMOS) and site-specific cancer risk in offspring born since 1992, followed up until diagnosis, death, or December 2018.**

| Cancer Type | PMOS/  No-PMOS | Events | Total | HR_crude | aHR*  Model 1 | aHR Model 2*** | aHR Model 3**** |
| --- | --- | --- | --- | --- | --- | --- | --- |
| Female genital organs # |  |  |  |  |  |  |  |
|  | No-PMOS | 28 | 22,726 | Ref | Ref | Ref | Ref |
|  | PMOS | 8 | 9,746 | 0.73 (0.33-1.62) | 0.72 (0.32-1.63) ** | 0.68 (0.29-1.56) | 0.68 (0.30-1.58) |
| Eye, brain and other parts of CNS |  |  |  |  |  |  |  |
|  | No-PMOS | 49 | 97,213 | Ref | Ref | Ref | Ref |
|  | PMOS | 16 | 21,526 | 1.49 (0.85-2.63) | 1.46 (0.82-2.61) | 1.57 (0.86-2.86) | 1.57 (0.86-2.85) |
| Lymphatic, hematopoietic  (excl. ALL) |  | | |  |  |  |  |
|  | No-PMOS | 26 | 97,213 | Ref | Ref | Ref | Ref |
|  | PMOS | 18 | 21,526 | **3.29 (1.79-6.05)** | **3.25 (1.75-6.02)** | **3.25 (1.65-6.43)** | **3.25 (1.65-6.43)** |
| Lymphatic leukemia up to 10 years |  |  |  |  |  |  |  |
|  | No-PMOS | 49 | 97,213 | Ref |  | Ref | Ref |
|  | PMOS | 11 | 21,526 | 1.02 (0.53-1.97) | 1.09 (0.55-2.15) | 1.06 (0.52-2.16) | 1.05 (0.51-2.14) |

Abbreviations= PMOS: Polyendocrine Metabolic Ovarian Syndrome; HR: Hazard ratio; aHR: adjusted Hazard ratio; CI: confidence intervals; CNS: Central Nervous system. Ref: reference.

* Model 1: Adjusted by sex of child, C-section, preterm birth (<37weeks) and Nordic countries. All models were stratified by the matching set.

** Model 1 additionally adjusted for C-section, preterm birth (<37weeks) and Nordic countries. All models were stratified by matching the set.

*** Model 2: Model 1 plus BMI.

**** Model 3: Model 1 plus BMI and any diabetes.

\# Only matching sets that contain female offspring in group of mother with PMOS and at least one female offspring in mothers without PMOS (male offspring were removed from the analysis).
